# Aetiology of thrombosed external haemorrhoids: a questionnaire study

**DOI:** 10.1186/1756-0500-2-216

**Published:** 2009-10-23

**Authors:** Ole Gebbensleben, York Hilger, Henning Rohde

**Affiliations:** 1 Park-Klinik Berlin-Weissensee, Innere Abteilung, Schönstrasse 80, 13086 Berlin, Germany; 2 Institut für Biostatistik, Bertoldstr. 1 - 3, 79098 Freiburg, Germany; 3 Praxis für Endoskopie und Proktologie, Viktoria-Luise-Platz 12, 10777 Berlin, Germany

## Abstract

**Background:**

It is important to better understand the aetiology of thrombosed external haemorrhoids (TEH) because recurrence rates are high, prophylaxis is unknown, and optimal therapy is highly debated.

**Findings:**

We conducted a questionnaire study of individuals with and without TEH. Aetiology was studied by comparison of answers to a questionnaire given to individuals with and without TEH concerning demography, history, and published aetiologic hypotheses. Participants were evaluated consecutively at our institution from March 2004 through August 2005.

One hundred forty-eight individuals were enrolled, including 72 patients with TEH and 76 individuals without TEH but with alternative diagnoses, such as a screening colonoscopy or colonic polyps. Out of 38 possible aetiologic factors evaluated, 20 showed no significant bivariate correlation to TEH and were no longer traced, and 16 factors showed a significant bivariate relationship to TEH. By multivariate analysis, six independent variables were found to predict TEH correctly in 79.1% of cases: age of 46 years or younger, use of excessive physical effort, and use of dry toilet paper combined with wet cleaning methods after defaecation were associated with a significantly higher risk of developing TEH; use of bathtub, use of the shower, and genital cleaning before sleep at least once a week were associated with a significantly lower risk of developing TEH.

**Conclusion:**

Six hypotheses on the causes of TEH have a high probability of being correct and should be considered in future studies on aetiology, prophylaxis, and therapy of TEH.

## Introduction

**Figure 1:**
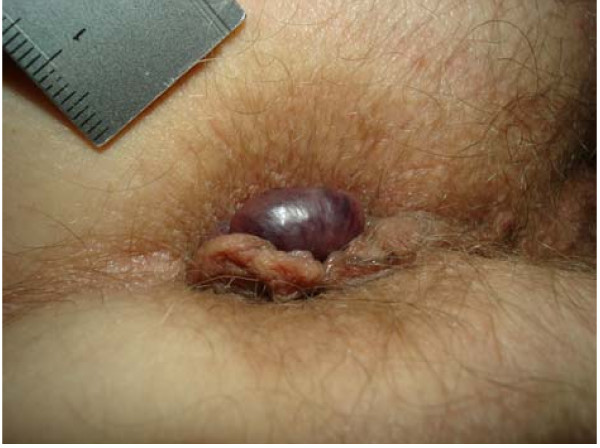
**Patient in knee chest postion, head left**. Thrombosed external haemorrhoid, diameter 10-15 mm, leftlateral of the anus with a dark subcutaneously lying clot in the middle and surrounding anal tags.

**Figure 2:**
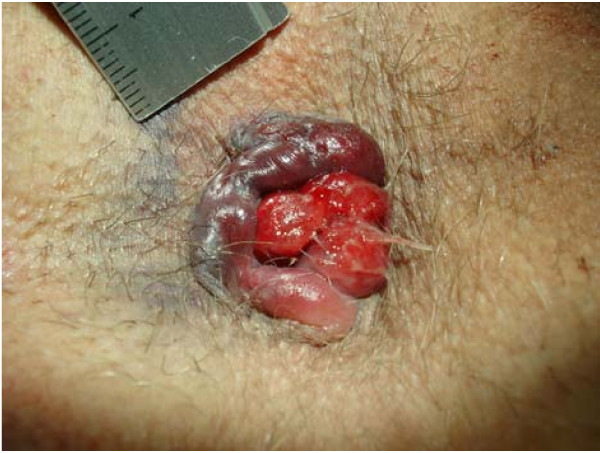
**Circular perianal thrombosis with bleeding into external haemorrhoids, oedema of concomitant anal tags leftlateral of the anus combined with prolapsing bright red haemorrhoids**.

**Figure 3:**
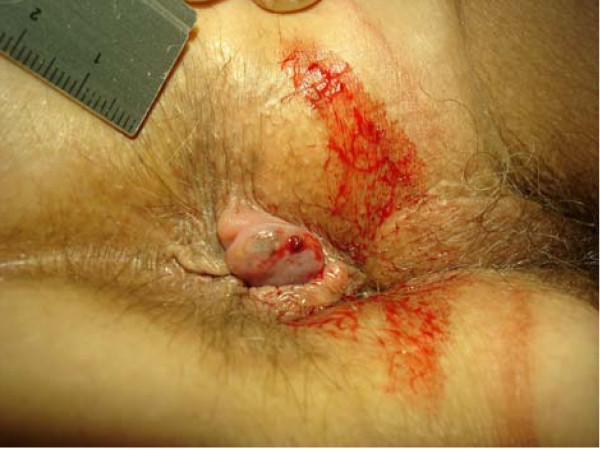
**Bleeding thrombosed external haemorrhoid (diameter 15 × 8 mm) rightlateral of the anus with concomitant tags**. A black clot is perforating the anal skin which is surrounded by fresh red blood.

**Figure 4:**
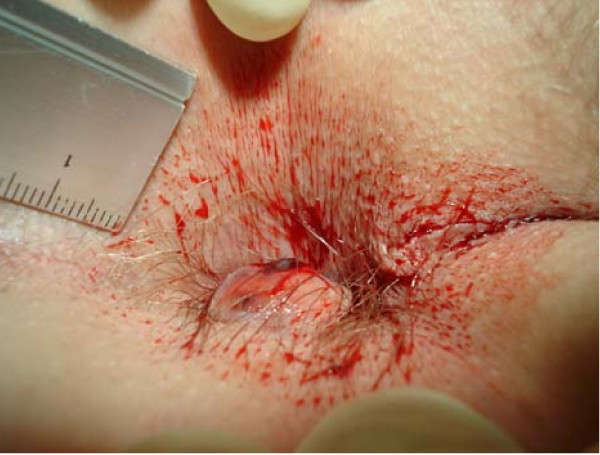
**Isolated leftlateral perforated and bleeding thrombosed external haemorrhoid with visible top of the subcutaneously lying clot (diameter 12 × 8 mm)**.

Although anorectal disorders are common [1-4] no data exist regarding the prevalence, incidence and aetiology of thrombosed external haemorrhoids (TEH) [3,5-9]. Common clinical presentations are as a single external pile (figure 1) or as circular thrombosis of external haemorrhoids (figure 2). A single TEH is characterized by acutely evolving, painful, circumscribed perianal swelling of a dark colour occasionally with bleeding (figure 3), and a perforating clot [2,5,6,10,11] (figure 4).

**Figure 5:**
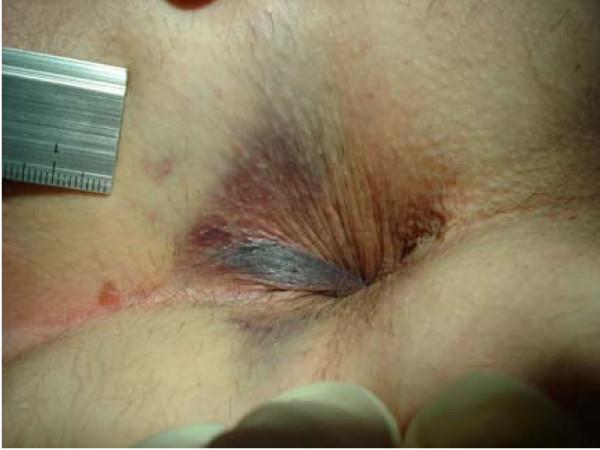
**Circular subcuteanously lying haematomas around the anus of a homosexual man after manipulations with a suction device to demonstrate the typical aspect of a subcutaneously lying haematoma**.

We limited our study to single lesions using Hancock's definition of "an acute localised thrombosis which may affect the external plexus" [2]. Synonyms of this clinical presentation include acute thrombosed external haemorrhoid [10,12], acute haemorrhoidal disease [13], anal haematoma [14,15], perianal haematoma [16,17], thrombosed haemorrhoid [18], haemorrhoidal thrombosis [15,19], and perianal thrombosis [20,21]. It has been shown that TEH is not a subcutaneous haematoma as it might happen in homosexual men (figure 5) but a thrombosis of external haemorrhoids [17]. Some authors therefore suggest to change the name of the disease to "perianal thrombosis" also in order to distinguish it from haemorrhoids since a causal connection between the two has never been demonstrated [20]. The goals of this study were to find published hypotheses for aetiology of TEH by a literature research, and to examine some of the putative causes of TEH using a questionnaire study presented to patients with and without TEH complaining of anal or abdominal symptoms.

## Methods

### Research literature

To gather data about TEH, we searched the MEDLINE database (December 1958 to January 2004) using the following keywords to find hypotheses about its pathophysiology: thrombosed external haemorrhoid, acute haemorrhoidal disease, perianal thrombosis, and thrombosed haemorrhoids.

One hundred eighty-seven papers were collected and consisted of reports that were therapeutic (44.4%), concerned with foreign objects (31.6%), reviews (20.8%) or case reports (3.2%). Titles and abstracts were reviewed and hard copies obtained for further examination. Additionally we reviewed journal reverence lists and standard textbooks and applied our existing knowledge of the primary publications in this area [11].

### Patients with and without TEH

Individuals of both genders, aged 16 - 80 years old, who entered consecutively our outpatient clinic from March 18 2004 to August 18 2005 were enrolled. Patients had been referred from general practitioners, physicians, urologists or gynaecologists for anal (i.e. bleeding, pain) and/or abdominal complaints (i.e. pain, flatulence). After proctologic assessment in the knee-chest-position [22], individuals underwent recto-, sigmoido- or colonoscopy if appropriate.

### Questionnaire

Both groups of participants (with and without TEH) were asked to complete our questionnaire (table 1) that focused on published hypotheses of TEH aetiology. Data from the questionnaires were collected, and the answers of patients with TEH were compared to those individuals without TEH.

**Table 1:** Questionnaire presented to the patients and hypotheses of TEH aetiology as published with reference numbers

Most of the questions are concerned with what happened to you the last days (Please tick the answer which fits most)	Published hypotheses of TEH aetiology as published with their reference numbers
Do you suppose to have haemorrhoids? (no/yes/I do not know)	[15,19,20,25,26]

Did you have an operation at your anus? (no/yes/I do not know)	[31]

Did you have diarrhoea? (no/yes/I do not know)	[24-26]

Did you take laxatives? (no/yes/I do not know)	[25]

Did you have hard stools? (no/yes/I do not know)	[24,25]

Did you strain at toilet during motions? (no/yes/I do not know)	[10,24,25,27]

Did you have cough and sneeze? (no/yes/I do not know)	[24,27]

Did you recently had a spicy meal? (no/yes/I do not know)	[27,28]

Did you recently had more alcohol than usual? (no/yes)	[27,28]

Did you sat on cold surfaces? (no/yes/I do not know)	[27]

Did you recently had an excessive physical effort? (no/yes/I do not know)	[29]

Did you recently lift a heavy load? (no/yes/I do not know)	[24-28]

Did you have sports, i.e. jogging? (no/yes/I do not know)	[27-29]

Did you have anoreceptive sex? (no/yes/I do not know)	[27]

Do you use dry toilet paper only after motions? (no/yes)	[23,31]

Do you use shower or wet wipes after motions? (no/yes)	[23,31]

Do you use the bathtub more than once a week? (no/yes)	[23,31]

Do you shower more than once or twice a week? (no/yes)	[23,31]

Do you clean your genitals before sleep more than once a week? (no/yes)	[23,31]

Do you use dry toilet paper after motions combined with wet cleaning? (no/yes)	[23,31]

Do you use gels/soaps after motions to clean your anus? (no/yes)	[23,31]

Do or did you have your menses? (no/yes)	[6,27,28]

Are you pregnant? (no/yes/I do not know)	[6,27,28]

Did you have childbirth within the last weeks? (no/yes)	[6,27,28]

### Statistics

At each step during the analysis we tried to enhance the significance of the statistical calculations. Initially we determined which of the 38 dichotomously coded variables (demographic, history) might have a significant bivariate relationship with TEH. To decipher this we used Fisher's exact test with a significance level of 5%. We than computed the odds ratio with a 95% confidence interval for each variable and searched for strong correlations.

A Pearson correlation coefficient above 0.36 was defined as strong. If a strong correlation between variables was found, only one of the correlating factors was retained. The factors of age, trainee, and retirement are naturally highly correlated. To prevent multivariate analysis from being biased by multi-colinearity, we decided to retain only one of these three variables. Age was considered to be the most general underlying factor and was therefore retained for further calculations. "Use of dry toilet paper exclusively" was also retained since it was determined to be a more concise fact than "Use of dry toilet paper combined with wet anal cleaning types". The strong correlation between these two variables justified the decision to keep only one for multivariate analysis.

For each of the remaining factors the variance inflation factor (VIF) was computed to ensure that multi-colinearity did not affect further evaluations. Finally a stepwise logistic regression analysis was performed to determine which of the remaining variables also had multivariate significance with TEH. For these analyses we used SPSS 15.0.1.1, 2007, SPSS Inc. Chicago, IL, USA.

## Consent

Our research study was carried out in compliance with the Helsinki Declaration.

Each patient gave written consent to participate in the study. They gave permission to take photos of their anal lesions to be presented in scientific medical journals or for medical educational purposes.

## Results

One hundred forty-eight individuals were enrolled: 72 patients with TEH only and 76 patients without TEH but with alternative diagnoses such as screening colonoscopy (22), stomach ache (21), IBD (9), colonic polyp (7), gastric ulcer (4), constipation (3), liver cirrhosis (1), cholelithiasis (1), pruritus ani (2), haemorrhoids (2), fissure-in-ano (2), rectal prolaps (1) or proctitis (1). Demographic variables in both groups of individuals were homogenously distributed (table 2).

**Table 2:** Demographic variables of patients with and without thrombosed external haemorrhoid (TEH)

	**Individuals with TEH** **(N = 72)**	**Individuals without TEH** **(N = 76)**	**p - values (t-test)**
Males (per cent)	61,1	60,5	

Age (mean +/- standard deviation, years)	42,7 (+/- 15,0)	49,4 (+/- 15,9)	p < 0.05

Body Mass Index (mean +/- standard deviation, kg)	24,3 (+/- 4,3)	25,5 (+/- 4,6)	p < 0.001

Non-Germans (N)	7	2	

Professions (per cent)			
employee	47,7	52,3	
worker	49,6	50,4	
civil servant	51,1	48,9	
housewife	48,2	51,8	
trainee	45,7	54,3	
pensioner	51,9	48,1	
self-employed	45,7	54,3	

Out of 38 possible aetiological factors 20 showed no significant bivariate correlation to TEH: gender, nationality, professions like employee, worker, housewife, and self-employed, the assumption to have haemorrhoids, previous anal surgery [23], diarrhoea [24-26], laxative use [25], hard bowels [24,26], straining at defaecation [10,24,25,27], sitting on cold surfaces [27], lifting a heavy load [24-28], coughing, sneezing [24,27], spicy meals [27,28], use of shower or wet wipes after defaecation [23], pregnancy [6,27,28], and current menses [28]. Since these variables were not significant, they were no longer traced throughout the remainder of the analyses.

The remaining 16 factors showed a significant bivariate relationship to TEH: age, careers as trainee, civil servant or retirement, participant in anoreceptive sex [27], engagement in excessive physical effort [29], engagement in sports [27,28], recent alcohol intake [27,28], frequency of bathtub use [23,30], frequency of shower use [23,31], frequency of genital cleaning before sleep [23,31], use of dry toilet paper after defaecation combined with wet cleaning [23,31], use of gels/soaps after defaecation [23,31], use of dry toilet paper only [23,31], and pregnancy [6,27,28].

After excluding three factors because of multi-colinearity and disregarding the female-specific factor of pregnancy, the remaining 12 potential risk factors proved to be suitable for overall multivariate analyses: age, BMI, civil servant, anoreceptive sexual activity [27], excessive physical effort [29], sports [27,28], recent alcohol intake [27,28], use of gel/soap after defaecation [23,31], use of dry toilet paper only [23,31], frequency of use of bathtub [23,31], frequency of use of shower [23,31], and cleaning genitals before sleep [31].

By multivariate analysis, six independent variables were found to be able to predict TEH correctly in 79.1% of cases. Three factors were associated with a significantly higher risk of developing TEH: individuals below the age of 46 years (p = 0.006, OR = 3.824 [1.468; 9.961]), excessive physical effort (P = 0.008, OR = 6.448 [1.622; 25.628]), and use of dry toilet paper combined with wet cleaning methods after defaecation (P = 0.007, OR = 3.785 [1.451; 9.875]). Three factors were found to be associated with a significantly lower risk for TEH: use of the bathtub (P = 0.015, OR = 0.259 [0.088; 0.767]), use of the shower (P = 0.001, OR = 0.036 [0.008; 0.154]), and cleaning genitals before sleep at least once a week (P = 0.001, OR = 0.184 [0.072; 0.470]).

A second multivariate analysis was performed using female patients only (N = 58) to determine whether pregnancy [6,27,28] is a significant multivariate risk factor. Upon analysis, it was determined that risk of TEH is not related to pregnancy. Sports [27-29] contribute to a high risk in the female population (P = 0.005, OR = 28.328 [2.745; 292.295]), whereas use of a bathtub (P = 0.021, OR = 0.081 [0.010; 0.683]), use of a shower (P = 0.041, OR = 0.102 [0.011; 0.912]), cleaning genitals before sleep at least once a week (P = 0.018, OR = 0.109 [0.017; 0.681]), and use of dry toilet paper after defaecation (P = 0.012, OR = 0.023 [0.001; 0.435]) were all demonstrated to decrease the risk of TEH.

## Discussion

As additional hypotheses regarding the aetiology of a disease are introduced, it becomes increasingly necessary to determine which risk factors are real and which are fiction. Numerous ideas about the aetiology of TEH have been published [6,10,19,23,25-28,32,33]. These hypotheses consist of both patient and physicians belief but few causes of TEH have been demonstrated in the literature [6,19]. It is important to better understand the aetiology of TEH because the recurrence rates are high [5,27,29], prophylaxis is unknown, and optimal therapy (conservative or surgical) is highly debated [8,10,34].

To better understand the risk factors for TEH, we initiated an explorative study encompassing a wide spectrum of demographic factors. One disadvantage of the study may be the small number of individuals used to gather data (148 in total). One advantage of the study is that the data was gathered prospectively unlike other available TEH studies, which are primarily retrospective analyses [3,5,7,8,10,16]. Out of the 38 possible factors related to TEH, six factors were able to correctly predict TEH in approximately 80% of individuals involved in the study. Some of these factors are common such as young age [5,6,10,30], while others are less common such as excessive physical effort [29]. The physical strain of excessive exercise is potentially comparable to straining with defaecation [10,24,25,27] because of hard stool [10,26] or sphincterspasm [33] which might lead to temporarily high intravenous pressure in the anal veins, possibly involving stretching and rupturing of the endothelial lining, initiating the thrombosis [32]. Some authors assume a causal connection between internal haemorrhoids and TEH [15,25,26] but this relationship remains unproven [22].

In females our multivariate analysis contrasted with data published by others [26-28] and showed no significant relationship of TEH to pregnancy, with the exception being during childbirth [6]. Similar to males, physical exertion such as sports were shown to be a positive risk factor for TEH (P = 0.005). Additionally, types of anal cleaning such as use of a bathtub, use of a shower, cleaning genitals before sleep, and use of dry toilet paper only after defecation all lower the risk of TEH.

Additional potential aetiologies of TEH include local irritation or inflammation of anal skin triggered by different agents like ointments [1,23,28], suppositories [28], anal fistulas [30], sitz baths [35], or detergents within soaps or shower gels [36]. Consistent use of dry and wet cleaning habits might be causative for TEH [31] as in our study, which leads to a significant high risk for developing TEH (P = 0.007). However we found data concerning the use of a bathtub (P = 0.015), use of a shower (P = 0.001), and cleaning genitals before sleep at least once a week (P = 0.001), all of which significantly lowered the risk. It seems likely that not one, but instead a spectrum of different factors may combine to contribute to initiating a TEH. In this study we were able to identify six factors with high correlative significance. These variables should be incorporated in future studies on the aetiology, therapy, and prophylaxis of TEH.

## Competing interests

There were no competing interests. The study was sponsored by the authors themselves. There were no financial or non-financial competing interests (political, personal, religious, ideological, academic, intellectual, commercial or any other).

## Authors' contributions

HR had the idea. All authors contributed to the design of the study and construction of the study protocol. OG was responsible for the research of literature. OG and HR saw the patients, and asked them to tick their answers into patients' questionnaire before proctologic assessment. Findings were ticked into a PC-documentation-sheet directly after medical assessment of each patient by OG and HR. Results were discussed with all authors. YH was responsible for statistical evaluations. OG wrote the first drafts of the paper which were revised by all authors. HR wrote the final draft.
